# A Study on the Reduction in Hydration Heat and Thermal Strain of Concrete with Addition of Sugarcane Bagasse Fiber

**DOI:** 10.3390/ma13133005

**Published:** 2020-07-06

**Authors:** Bruno Ribeiro, Takashi Yamamoto, Yosuke Yamashiki

**Affiliations:** 1 Graduate School of Advanced Integrated Studies in Human Survivability, Kyoto University, Kyoto 606-8501, Japan; yamashiki.yosuke.3u@kyoto-u.ac.jp; 2 Department of Civil and Earth Resources Engineering, Graduate School of Engineering, Kyoto University, Kyoto 606-8501, Japan; yamamoto.takashi.6u@kyoto-u.ac.jp

**Keywords:** sugarcane bagasse fibers, sugarcane bagasse ash, fresh properties, heat of hydration, thermal strain, mechanical properties

## Abstract

Early prevention methods in massive concrete structures to control the heat of hydration and, consequently, the development of cracks due to thermal expansion are important subjects, since these cracks may compromise structural integrity. In the present study, the sugarcane residues in massive concrete were used in order to investigate the reduction in the heat of hydration, the thermal expansion resistance, and also the fresh and mechanical properties of the concrete. The results showed that, by adding 2.0% of bagasse fiber and 5.0% of pozzolanic material to the concrete, the heat of hydration was reduced, and the strain due to the thermal expansion was smaller than the control mixture. Moreover, the compressive, flexural, and split tensile strength increased in comparison to the control mixture.

## 1. Introduction

The reaction between water and cement in massive concrete structures like dams, pavements and piers produces heat and increases the temperature of the concrete [1,2]. During the cement hydration, the internal part of the structure will reach a higher temperature than the surface, and excessive differences in the temperature may form cracks in the structure. Cracks may form when the thermal stresses exceed the tensile strength of the young concrete, arise as a result of concrete shrinkage, and/or during the service of the structure, for example, changes in the temperature of the concrete due to the climate changes and vibrations externally generated. Moreover, the cracks tend to develop where the concrete is lower quality, excessively porous due to the separation of the concrete mixture components or its insufficient vibration or because the larger aggregate particles were not entirely coated with the concrete mixture [3,4]. Some special methods are required to control the temperatures during cement hydration. Therefore, some actions should be performed before, during, and after concrete placement to prevent the heat of hydration formation emanating from the center to the surface of the concrete [5].

Several previous researchers have tried to reduce hydration heat by replacing a certain amount of cement with pozzolanic materials, such as silica fume, fly ash, and oil palm fuel ash. Their results show that, when a certain amount of these materials is used in place of cement, it is possible to decrease the temperature of the concrete [6,7,8].

Nili et al. investigated the effects of supplementary cementitious materials on the temperature rising profile, heat evolution and early-age strength development of medium- and high-strength concrete. A total of 13 different mixtures were prepared, with two water-cement ratios (0.3 and 0.46). Natural pozzolan, fly ash, and silica fume were included in the specimens. The results showed that natural pozzolan, particularly fly ash served to decrease the amplitude of peak temperature, delay the occurrence of the peak, and decrease the sharpness of the temperature rising profiles [9].

Awal et al. investigated the performance behavior of palm oil fuel ash (POFA) in reducing the heat of hydration of concrete. Two concrete mixes, namely OPC (ordinary Portland cement) concrete, i.e., concrete with 100% OPC as control, and POFA concrete, i.e., concrete with 30% POFA and 70% OPC were prepared, and the temperature rise due to heat of hydration in both the mixes was recorded. It has been found that palm oil fuel ash not only reduced the total temperature rise, but also delayed the time at which the peak temperature occurred. The results obtained and the observations made clearly demonstrate that the partial replacement of cement by palm oil fuel ash is advantageous, particularly for mass concrete where thermal cracking due to excessive heat rise is of great concern [10].

Apart from the use of pozzolanic materials, there are several countermeasures that can improve thermal crack resistance and decrease the effect of the strain shrinkage of the concrete [11]. The utilization of fibers is one of these countermeasures. The major advantage of fiber reinforcement is to impart additional energy-absorbing capability by transferring a brittle material into a pseudoductile one [12].

Sarabi et al. used waste turnery steel fibers in massive concrete in order to control the generated cement hydration heat and, consequently, the potential of cracking due to the thermal expansion. The amount of cement used was reduced without changing the compressive strength. By substituting a part of the cement with waste steel fibers, the costs and the generated hydration heat were reduced, and the tensile strength increased. The results showed that, by using 0.5% turnery waste steel fibers and, consequently, by reducing to 32% the cement content, the hydration heat was reduced to 23.4%, without changing the compressive strength. Moreover, the maximum heat gradient was reduced from 18.5% in the plain concrete sample to 12% in the fiber-reinforced concrete sample [13].

Recently, the application of agro-residues in concrete has gained interest due to the high amount of these materials available throughout the world, and also due to environmental issues such as the incorrect disposal of these residues. Among several types of agro-residues, there is the bagasse, which is a fibrous residue of the sugarcane [14]. Natural fiber-reinforced cement composites have gained increasing interest to researchers and manufacturers seeking to improve construction materials. Due to their high performance in mechanical properties and low cost, natural fiber-reinforced cement composites have a high potential for replacing standard fiber materials [15,16,17,18].

The sugarcane bagasse is mostly composed of cellulose, hemicellulose, and lignin. Cellulose, hemicelluloses, and lignin are in the family of polysaccharides, and these polysaccharides are composed of various types of sugars. Therefore, these sugars act as setting and hydration retarding agents [12]. The combination of both pozzolanic materials and bagasse fibers to decrease the heat of hydration and control the generation of cracking due to the thermal expansion remains unclear. In the present research, sugarcane residues were used in place of sand in order to investigate the behavior of the cement hydration heat and, consequently, to evaluate the potential resistance these residue materials have against the generation of cracks due to thermal expansion in massive concrete.

## 2. Methodology

### 2.1. Preliminary Trials Applied to Sugarcane Bagasse Residues

**Figure 1:**
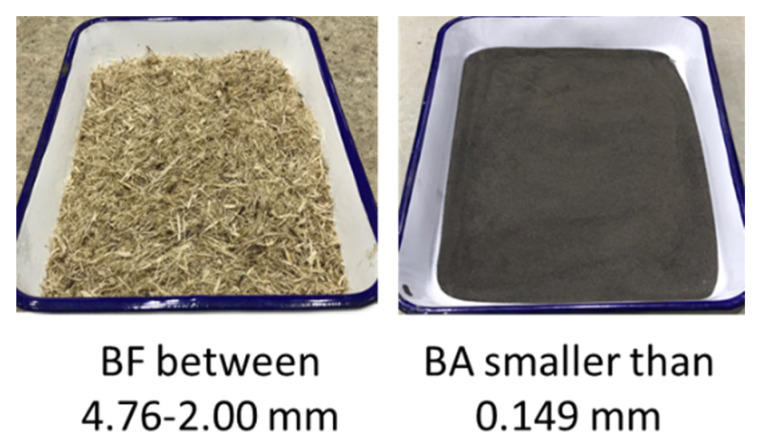
Categorization of sugarcane residual materials.

The sugarcane residues (raw bagasse and burned residues) were acquired from a sugar mill in Okinawa Prefecture, Japan. In the case of the raw bagasse, it was dipped in water at 30 °C for 30 min and then dried in the open air for 14 days. The intent of this process was to reduce the residual sugar content of the bagasse and eliminate impurities [19,20,21]. Afterward, the raw bagasse was sieved. The bagasse fibers (BF) used in this study were passed through a 4.76 mm sieve and remained in a 2 mm sieve with a length variation between 8 and 44 mm. Prior to the preparation of specimens, the bagasse fibers were treated with an alkali solution to improve the strength of fibers against the alkaline attack [21,22]. The bagasse fibers were soaked in a solution of 5% Ca(OH)_2_ for 24 h [23,24]. In the case of the burned residues, it was not washed in order to avoid small particles of ash coming into contact with water. The burned residues that passed through a 0.149 mm sieve were classified as bagasse ash (BA). Figure 1 shows the categorized residual materials that were used in this study.

### 2.2. Materials

The specimens were made using ordinary Portland cement (C). The fine aggregates (S) and the coarse aggregates (G) were acquired from manufacturer, Nishijima, Hyogo, Japan. In order to comparisons, a commercially available fly ash (FA) grade II was used. The physical properties of these materials are given in Table 1.

For the concrete mixture, two kinds of admixtures were used in this study to satisfy the requirements of the fresh concrete. The properties of the water-reducing agent (WRA) and air-entraining agent (AEA) are shown in Table 2 and the characteristics of the bagasse fibers are shown in Table 3.

### 2.3. Concrete Mixture

The mix proportions of the concrete are shown in Table 4. Mixture C was prepared in the laboratory, with a water to binder ratio (W/B) of 45%. Mixture C represents the control specimens and contains no sugarcane residue materials. In the case of BF specimens, two mixtures were prepared with bagasse fiber volume ratios of 2.0% (BF2) and 5.0% (BF5) in comparison to the total mixture volume. In addition, specimens were made using bagasse ash (BA) and fly ash (FA). In these cases, the volume ratio of the bagasse ash and fly ash was 5.0% in comparison to the total volume of sand. Note that, in the case of BA and FA, the specimens were prepared with the same volume of fibers as BF2, and the bagasse ash and fly ash acted as a binder. Thus, the W/B becomes 43% and 41%, respectively. In all cases, the fibers were replaced by sand. The maximum size of the coarse aggregate (G_max_) was 15 mm.

### 2.4. Preparation of Concrete Specimens and the Tests Applied

For each concrete mixture, specimens of Φ100 mm × 200 mm were cast in order to determine the compressive strength (JIS A 1108), elasticity modulus (JIS A 1149), split tensile strength (JIS A 1113), porosity rate and water retention rate of concrete. Furthermore, specimens of 100 mm × 100 mm × 400 mm were prepared to determine the flexural strength (JIS A 1106). Note that three specimens were used for each test.

The porosity rate test was based on the mass of the specimen. First, the specimens were completely dried in a drying furnace at 105 °C for 24 h. Afterwards, the specimens were stored in a room at 20 °C and relative humidity of 60% until the temperature of the specimen decreased and became equal to the ambient temperature, then the mass of the specimen was weighed. Subsequently, the specimens were immersed in water at 20 °C for 48 h, then the surface-dried mass of the specimen was weighed. The porosity rate was determined by the following equation:

(1) $$\varepsilon =\frac{W_{s}-W_{d}}{\rho \times V}\times 100$$

where ε: porosity rate (%);

$W_{d}$: mass of absolutely dried specimen (g);

$W_{s}$: mass of surface-dried specimen (g);

ρ: density of water (g/cm^3^);

V: volume of specimen (cm^3^).

After the porosity rate test, the moistened specimens were placed in a room at 20 °C and relative humidity of 60%, and the mass change of specimen was recorded every 24 h for 7 days. This mass change was determined as water retention rate.

**Figure 2:**
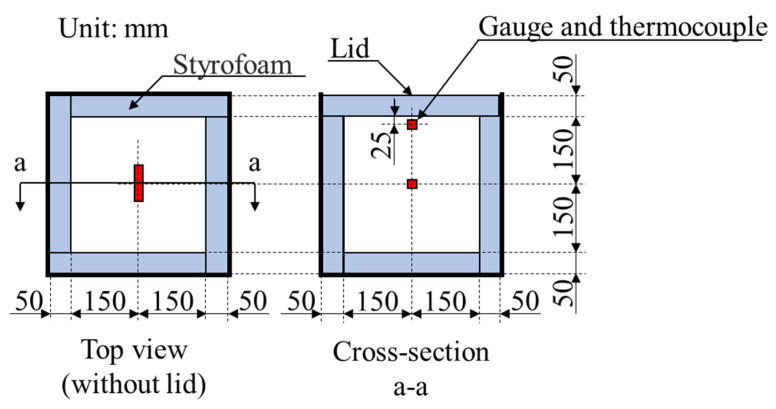
Outline of massive concrete specimen.

In addition, in order to simulate a massive concrete structure, 2 specimens of 300 mm × 300 mm × 300 mm of each concrete mixture were prepared. The temperature and the strain of the concrete were measured by two thermocouples (T-G-0.65) and two mold strain gauges (PMFL-60) embedded in concrete, respectively. The thermocouples and the gauges were settled at the surface (cover thickness of 20 ± 2 mm) and the center (cover thickness of 145 ± 2 mm), as shown in Figure 2.

All the concrete mixtures were mixed using a laboratory pan-type mixer. First, the coarse aggregate, cement/ashes, and fine aggregate were put in the mixer and mixed for 30 s. Next, the water/fibers were put in the mixer and mixed for an additional 120 s. Before casting, the slump flow and the air content were conducted on the concrete mixture to determine its workability.

After the casting, all specimens were cured in the laboratory at 20 °C for 24 h. The specimens were then de-molded and cured underwater, at 20 °C for 28 days. However, the massive concrete specimens were not de-molded and were instead cured in the air at a room temperature of 20 °C and 60% RH for 28 days. During the curing period, the temperature and the strain due to the thermal expansion of concrete were measured by using thermocouples and gauges, as shown in Figure 2.

## 3. Results and Discussions

### 3.1. Fresh Concrete

**Figure 3:**
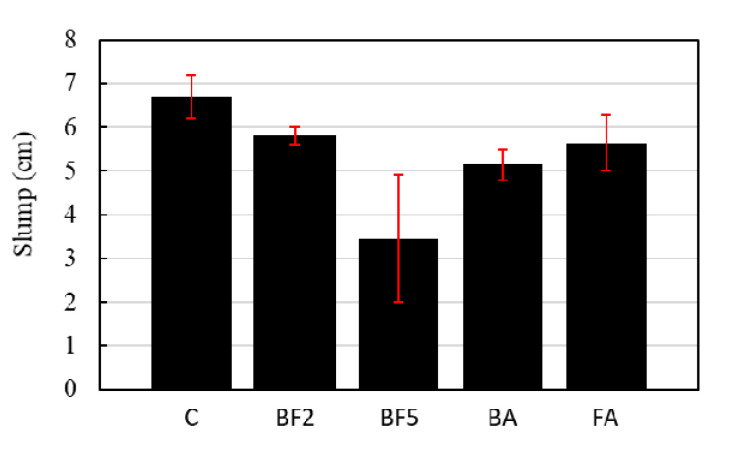
Slump test results.

**Figure 4:**
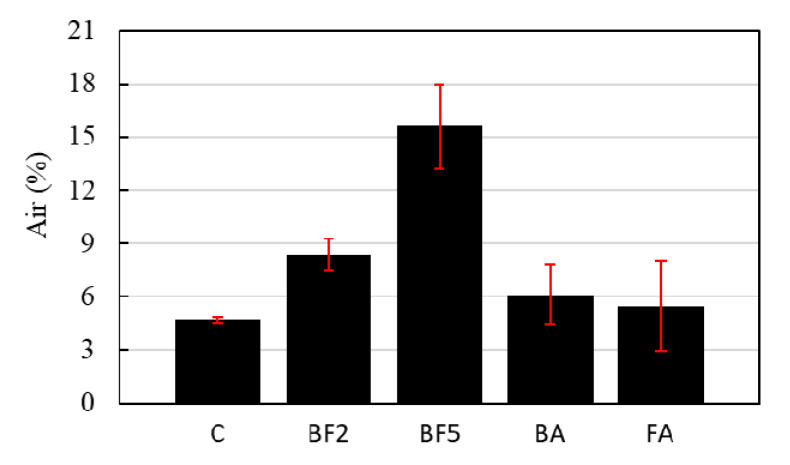
Air content test results.

The slump test and air content test results are shown in Figure 3 and Figure 4, respectively. As shown in Figure 3, the slump of concrete decreased with the addition of the bagasse fiber. The slump of the control concrete is 6.7 cm, while in the cases of BF2, BA, and FA which the fraction volume of the fiber was 2%, the slump reduced to 5.8, 5.2, and 5.7 cm, respectively. In the case of BF5, where 5% of the volume was bagasse fiber, the slump decreased to 3.5 cm. This decrease is because of the formation of a bridge network of fibers that restrained the deformation of the fresh concrete during the test. As a result, the more bagasse fiber is added to the concrete mix, the greater the fiber network is, which decreases the concrete workability. Another factor that contributed to this decrease is the fact that a certain amount of water may have been absorbed by the bagasse fiber during the mixture, due to the hydrophily of bagasse fiber [25,26]. In the cases of BA and FA, with the addition of ashes and the increase in the binder amount, the viscosity of concrete increased, resulting in a brief decrease in the slump when compared to BF2.

The air content in the case of the concrete without fibers is about 4.7%. However, the amount of air increases as the amount of mixed fibers increases. For the cases of BF2, BF5, BA, and FA, the air content values obtained were 8.4, 15.6, 6.1, and 5.5%, respectively. The rise in the air content with the addition of bagasse fiber can be explained due to fine bubbles of air clumping together while the concrete mixes; consequently, the amount of empty space may increase.

Figure 4 further reveals that the air content of the cases of BA and FA, which contain 5% of pozzolanic materials, is lower than that of BF2. These results are a consequence of the filler effect: due to the replacement of sand with ashes, the ashes occupy the micro and macro empty spaces that were created during the mixing.

### 3.2. Heat of Hydration

**Figure 5:**
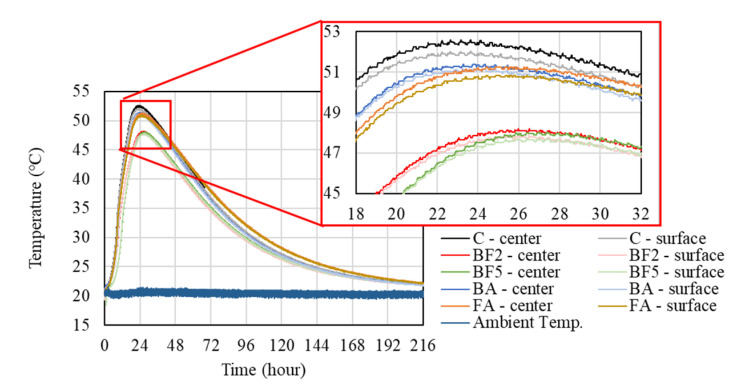
Heat of hydration of cement.

The plot of temperature versus time for the different mixtures and the ambient temperature can be seen in Figure 5.

As Figure 5 illustrates, the peak of heat of hydration in the center of the specimens in the case of the control mixture was nearly 52.5 °C, resulting in the highest temperature reached in this study. In the cases of BF2, BF5, BA, and FA, this value decreases to around 48.3, 48.0, 51.4, and 51.3 °C, respectively. It can be concluded from Figure 5 that, the hydration heat of all mixtures containing bagasse fiber was reduced. These findings are consistent with previous studies, which indicate that the composites (cement paste) made with components of bagasse exhibit setting temperatures that are lower than the reference [27]. According to Bilba et al. [27], there are two phenomena that can explain this temperature decrease: the reaction between water and cement, which is exothermic, and the reactions between water and components of bagasse fiber, which are endothermic.

In the case of BF2 and BF5, the difference in the peak temperature of the heat of hydration was around 0.3 °C. As the amount of fibers increases in the case of BF5, a high number of empty spaces between the fiber and the matrix are created, reducing the thermal conductivity. Therefore, the absorption of the hydration heat by the surrounded fiber components becomes difficult, resulting in the same temperature as BF2.

In the cases where the bagasse ash and the fly ash were added into the mixture to replace the sand, the temperature was approximately 1 °C lower compared to the case of C; however, the temperature was about 3 °C higher than for those mixtures where bagasse fibers were added. The reason for this increase in temperature in the cases of BA and FA, compared to BF2 and BF5, is due to the difference in the water to binder ratio. As shown in Table 4, the W/B of BA and FA is 43% and 41%, respectively, or 2 or 4% smaller than the other cases.

Furthermore, from Figure 5, it can be observed that, in the mixtures with the bagasse fiber, the peak temperature is reached later than C, which was around 22:30 h after the placement. For BF2, BF5, BA, and FA, the temperature peak was achieved, respectively, 26:00, 27:00, 24:10, and 25:10 h after the concrete placement. One probable explanation is that the presence of some water-soluble sugars may have retarded the setting of concrete [27]. In order to verify the presence of sugar, 4 g of bagasse fiber (BF) was cut and 40 mL of H_2_O was added, subsequently, it was allowed to stand for about 2 h. Later, this mixture was centrifuged at 8000 rpm at 20 °C for 30 min. Then, 10 mL supernatant was lyophilized and dissolved in 1 mL of acetonitrile/water (CH_3_CN/H_2_O = 75/25). This solution was used as a sample to analyze the reducing sugar. Afterward, 0.2 mL of sample, 0.4 mL of reagent A (NaCO_3_ (40 g/L), Glycine (16 g/L), CuSO_4_·5 H_2_O (0.45 g/L)) and 0.4 mL of reagent B were mixed. The mixture was heated at 100 °C for 12 min and rapidly cooled. Then, 1 mL of H_2_O was added, and the absorbance at 450 nm was measured. From the calibration curve prepared with glucose, the reducing sugar content of the sample was determined as a glucose equivalent. This result indicates that the reducing sugar content was 1.05 mg/g, confirming the presence of water-soluble sugars that affected the hardening and hydration of cement. These soluble sugars may form a protective layer around the hydrating cement [28], preventing water from percolating to further hydrate the cement grain [12,29]. As a consequence, the heat of hydration was reduced, and the setting of the concrete was retarded.

Moreover, Figure 5 reveals a difference between the internal and surficial temperature. The peaks of the surface temperature (cover 2 cm) for C, BF2, BF5, BA, and FA were approximately 52, 47.9, 47.8, 51.2, and 50.8 °C, respectively. In the case of C without the sugarcane bagasse fiber, the difference in the surface and the center temperature achieves a value of about 0.5 °C. In the other mixtures with the bagasse fiber, this value varies between 0.2 and 0.3 °C. Usually, the difference between the internal and surficial temperature is notably higher, because the concrete surface is influenced by the ambient temperature [5,30,31]. However, the reason why the difference between internal and surficial temperature obtained in this research was not higher is due to the fact that the Styrofoam, as the thermal insulation material, completely isolated the concrete from the outside temperature influence.

### 3.3. Relationship between the Heat of Hydration and Strain

**Figure 6:**
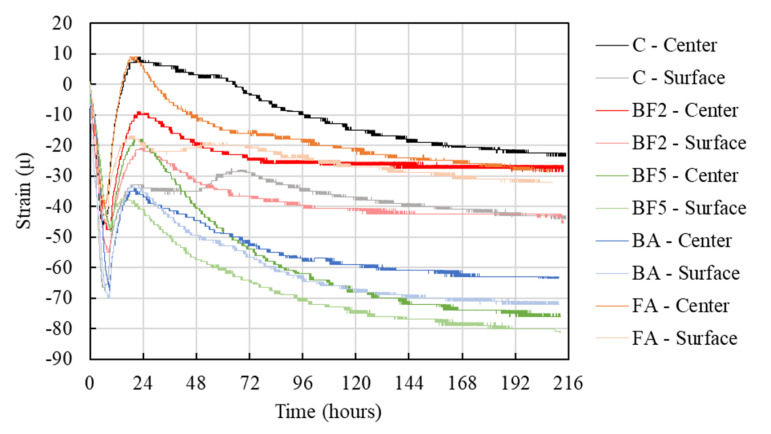
Comparison of the strain of each mixture.

The relationship between the heat of hydration and the strain on each mixture is shown in Figure 6 (shrinkage (-), expansion (+)). As can be seen in Figure 6, after the concrete placement, the strain gauges are pressed initially. After that, the concrete tends to expand due to the increase in the temperature as a consequence of the heat of hydration.

**Figure 7:**
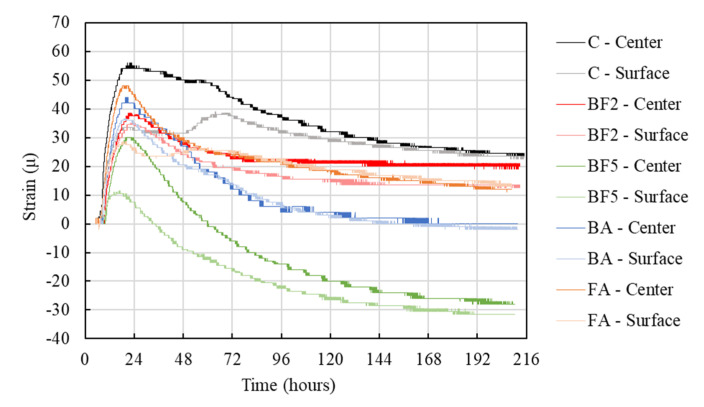
Thermal expansion.

The strain due to the thermal expansion of each mixture is shown in Figure 7. Note that the initial strain value was set right after the thermal expansion began. As Figure 7 shows, in the case of C, the strain rose to a value of approximately 55 μ, while in the cases of BF2, BF5, BA, and FA, the strain achieved 39, 30, 44, and 48 μ, respectively. When bagasse fiber is added to the mixture there, is a tendency to decrease the strain of the concrete. As well as the increase in the temperature of the concrete, which can influence the strain, the fibers may have reduced the expansion due to the transference of stress among the network of bagasse fiber bridges to other cross-sections.

Furthermore, Figure 7 shows that the strain on the specimen surface is lower than the strain in the center. This is due to the fact that the strain gauge used in this study measured only the horizontal direction (see Figure 3), and we also only used one gauge in the center and one on the surface (cover 25 mm); therefore, the measurement of the strain in other directions was not able to be measured. Although the surface and center temperatures are almost the same, the vertical strain may have been more affected than the horizontal strain on the surface of the concrete. However, supplementary studies should be done in the future to measure the strain in different directions to clarify this point.

### 3.4. Porosity Rate and the Water Retention Rate

**Figure 8:**
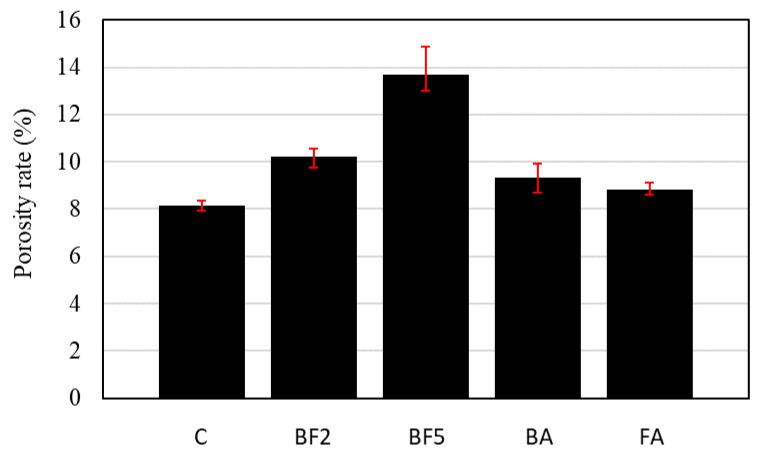
Porosity rate of each concrete mixture.

Figure 8 shows the porosity rate test results.

As shown in Figure 8, the porosity rate increased as the replacement rate of bagasse fiber increased. This is because a higher amount of entrapped air in the mixture with bagasse fibers is created during the mixing than in that without bagasse fibers.

However, in the cases of BA and FA, where the ash was added, the porosity rate of the concrete decreases in comparison to the case of BF2, which contains the same volume of fiber. Although the solid content per cubic meter of BA and FA is higher than the solid content of BF2, the reason for this may also be related to the filler effect of the ash. When adding ashes to concrete mixtures, the ashes act to filling the voids and reduce the number of pores and probable microcracks of the concrete during pozzolanic reactions.

**Figure 9:**
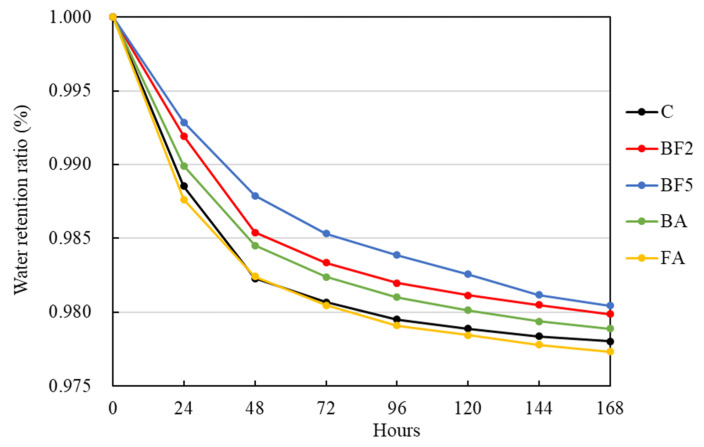
Water retention rate of each mixture.

The water retention rate of each concrete mixture is shown in Figure 9.

As shown in Figure 9, the water retention ratio of the mixtures in which fibers were added increased when compared to the control mixture. It is also possible to see that the higher the amount of fiber added, the higher the water retention in the specimen is. The high retention of water in BF5 is due to the high percentage of fibers used and due to the characteristics of bagasse fiber, which has a high capacity for the absorption and retention of water.

However, after 120 h, the water retention rate of BF5 decreases to nearly the same values as BF2. The natural fibers are very hydrophilic [25,26]. For this reason, the wetness of the whole specimen tends to be balanced, transferring the water from the center of the specimen to the surface, since the surface of the specimen is more affected by natural drying. The higher amount of fiber tends to retain more water in certain periods. However, due to the amount of fiber, nearly all of the specimen surfaces is are in the case of BF5 and. consequently, are more affected due to natural drying, as the water may have been transferred by the fiber network faster than the other cases to keep the wetness of the specimen balanced, resulting in the decrease after 120 h.

In cases when 5% of ashes were added, the water retention ratio is smaller than the BF2 case, even though these cases contain 2% of fibers added in the mixture. In particular, in the case of FA, the water retention was smaller than the control mixture. This reduction may be related to the pozzolanic reaction of the ashes, in which the water has been consumed by the fibers.

### 3.5. Compressive Strength

**Figure 10:**
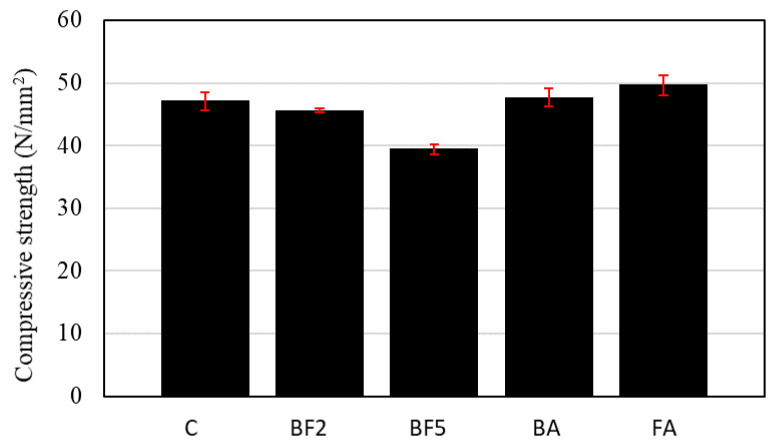
Compressive strength of each concrete mixture.

Figure 10 illustrates the compressive strength of each concrete mixture. From Figure 10, it is possible to see that, with the addition of 2% of bagasse fiber, the compressive strength briefly decreases, probably due to the increase in the porosity of the concrete. In the case of BF5, which resulted in a higher porosity, the compressive strength decreases by about 16% in comparison to C. Since the entrapped air tends to increase during the mixing and decrease the compressive strength, the use of proper vibration techniques can be helpful in removing entrapped air.

On the other hand, Figure 10 demonstrates that the compressive strength increased in the cases of BA and FA where the ashes were added into the mixtures. Usually, the low reactivity and extremely slow reaction rate of fly ash at ambient temperatures leads to the low strength of the concrete at an early age [32,33,34]. However, the increase in the compressive strength in the cases of BA and FA is due to the fact that, when the ashes are added into the mixture, the amount of powder used increases the binder amount, as shown in Table 4. Another probable reason may be due to the alkali activation, which effectively accelerates the pozzolanic reaction of ash [34]. Since the fibers were treated with a solution of 5% Ca(OH)_2_ for 24 h, the internal alkali solution of the fibers may have been activated on the surface of the ash particles at an early age, accelerating the pozzolanic reaction during the water immersion curing period.

### 3.6. Modulus of Elasticity

**Figure 11:**
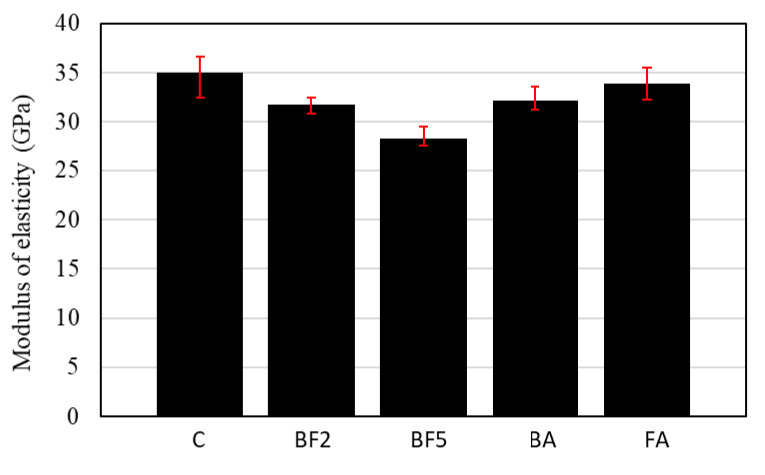
Modulus of elasticity of each mixture.

Figure 11 shows the elasticity modulus of the concrete. As illustrated in Figure 11, the moduli of elasticity for C, BF2, BF5, BA, and FA were 35.0, 31.8, 28.3, 32.1, and 33.9 GPa, respectively. From these results, it is possible to conclude that the modulus of elasticity of the concrete decreases with the addition of fiber, which gains ductility in comparison to C.

According to the Japan Society of Civil Engineers (JSCE) [35], in the case of ordinary concrete with a compressive strength of 40–50 N/mm^2^, the elastic modulus is in the range of 31–33 GPa. Although the modulus of elasticity in the case of C and FA is slightly higher than this range, in the case of BF5, the elastic modulus decreases drastically to around 28 GPa. This is due to the high amount of fiber added to the mixture, which increased the porosity and reduced the modulus of elasticity.

### 3.7. Flexural Strength

**Figure 12:**
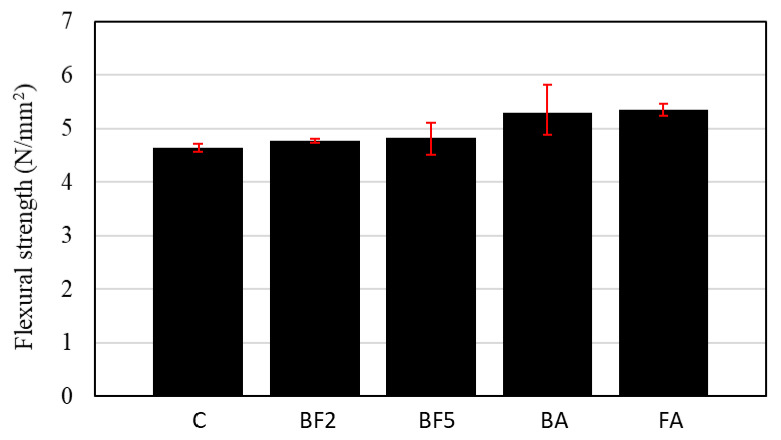
Flexural strength of each concrete mixture.

Figure 12 shows the flexural strength of each concrete mixture. As can be seen in Figure 12, the flexural strength was 4.63, 4.77, 4.83, 5.30, and 5.35 N/mm^2^ for C, BF2, BF5, BA, and FA, respectively. From these results, it can be concluded that the higher the amount of fiber added, the higher the acquired flexural strength is. However, in the case of BF5, the flexural strength was not proportional, resulting in a flexural strength near to that of BF2. This tendency indicates that, when a high amount of fibers are added into concrete, these fibers may not effectively transfer the stress to the other cross-sections. This may be due to the fact that, with the addition of a high amount of fibers, the concrete density tends to decrease and, consequently, the porosity tends to increase. Therefore, the high number of interfacial voids between the fiber and the matrix may increase, affecting the transmission of stress.

In addition, Figure 12 shows that, in comparison to BF2, the flexural strength increased when the ashes are added into the mixture. This increase is related to the pozzolanic reaction. The pozzolanic reaction leads to the formation of additional C–S–H gel [36], which may have filled up the interfacial voids between the fiber and the matrix. Consequently, the flexural strength increases as the fiber/matrix interface contact increases, allowing for the homogeneous distribution of stress to other cross-sectional parts of the specimens.

### 3.8. Split Tensile Strength

**Figure 13:**
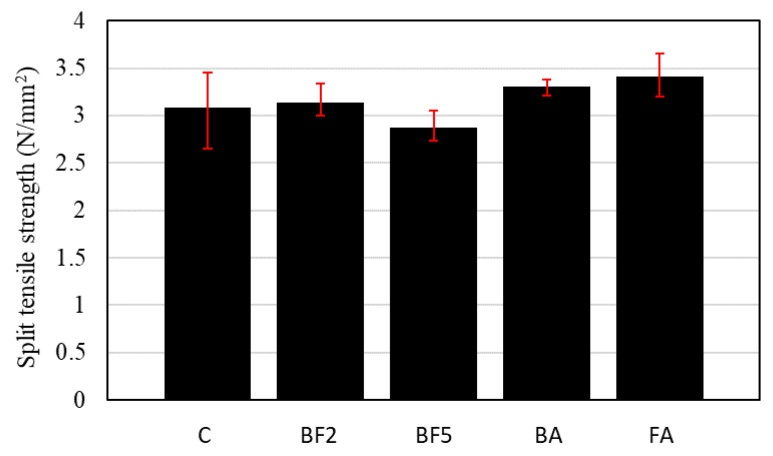
Split tensile strength of each concrete mixture.

Figure 13 shows the results of the split tensile strength test.

From Figure 13, it is observed that the 28-day split tensile strength increased when 2.0% of fiber content was added to the mixture. This increase was more significant when 5% of ashes were added to the mixture. In the case of BA and FA, the split tensile strength was 3.30 and 3.41 N/mm^2^, respectively.

However, when 5% of fiber was added, the split tensile strength decreased to around 2.87 N/mm^2^, approximately 7% lower in comparison to the control mixture. Due to the fact that the tensile failure section is limited to the one in the tensile splitting test, the effect of fiber bridging to redistribute the stress to another cross-section is limited, a phenomenon that is even more pronounced due to the high amount of voids in the fiber/matrix interface.

## 4. Conclusions

The use of sugarcane residues to replace sand may be a way to make low-cost and environmentally friendly materials. In addition, their use in concrete could reduce the heat of hydration and the strain due to the thermal expansion, which may be a countermeasure against the generation of cracks in massive concrete.

The results obtained in this study can be summarized as follows:

1. The slump decreased with the addition of the fiber amount compared to the control mixture. However, the amount of air increased as the amount of mixed fibers increased.
2. With the addition of the bagasse fiber, the heat of hydration of all mixtures was reduced. In the case when 5% of the bagasse fiber was added, the peak temperature neared 48 °C, approximately 4.5 °C lower than the control mixture.
3. In the cases in which the bagasse fiber was added, the peak temperature was reached later than the control mixture. In the case when 2% of the bagasse fiber was added, the temperature peak was achieved 26 h after the concrete placement, while the control mixture temperature peak was achieved 4 h earlier.
4. In the case of the control mixture, the strain rose to a value of approximately 55 μ, while, in the case of the mixture in which 5% of bagasse fiber was added, the strain value was 30 μ, a difference of about 25 μ.
5. In the case where the bagasse fiber was added, the compressive strength decreased. However, the compressive strength increased when the ashes were added to the mixtures, thereby exceeding the control mixture.
6. The flexural strength of all concrete specimens with added fiber exceeded the value of the control specimen.
7. The split tensile strength increased when 2.0% of the fiber content was added to the mixture. On the other hand, with 5.0% of the fiber, the split tensile strength decreased.

## Figures and Tables

**Table 1:** Physical properties of cement and aggregates.

Properties	Materials				
	Cement	S	G	BA	FA (II)
Density (g/cm^3^)	3.16	2.57	2.57	2.1	2.24
Total alkali content (%)	0.56	―	―	―	―

**Table 2:** Properties of admixtures.

Admixtures	Main Components	Color	Density	Total Alkali	Cl^−^
			(20 °C, g/cm^3^)	Content (%)	Content (%)
WRA	Complexes of lignin	Dark	1.23–1.27	1	0.03
(No.70)	sulfonic acid compound	brown			
	and polyol				
AEA	Alkyl ether type	Light	1.02–1.06	1.1	0.01
(303A)	anionic surfactant	yellow			

**Table 3:** Characteristics of the bagasse fibers.

Properties	BF
Density (g/cm^3^)	0.71
Length (mm)	17.9 (average)
Diameter (mm)	0.56 (average)
Aspect Ratio	32
Tensile strength (N/mm^2^)	132

**Table 4:** Mix proportions and fresh property of the concrete specimens.

Composites	Fiber	W/B	Unit (kg/m^3^)								
	(Vol. %)	(%)	C	W	S	G	BA	FA	BF	WRA	AEA
C	—	45	389	175	876	950	—	—	—	1.17	0.0125
BF2	2.0				824		—	—	14		
BF5	5.0				746		—	—	36		
BA	2.0	43			780		22	—	14		
FA	2.0	41					—	39			
